# Physically inspired deep learning of molecular excitations and photoemission spectra†

**DOI:** 10.1039/d1sc01542g

**Published:** 2021-06-30

**Authors:** Julia Westermayr, Reinhard J. Maurer

**Affiliations:** Department of Chemistry, University of Warwick Gibbet Hill Road Coventry CV4 7AL UK r.maurer@warwick.ac.uk

## Abstract

Modern functional materials consist of large molecular building blocks with significant chemical complexity which limits spectroscopic property prediction with accurate first-principles methods. Consequently, a targeted design of materials with tailored optoelectronic properties by high-throughput screening is bound to fail without efficient methods to predict molecular excited-state properties across chemical space. In this work, we present a deep neural network that predicts charged quasiparticle excitations for large and complex organic molecules with a rich elemental diversity and a size well out of reach of accurate many body perturbation theory calculations. The model exploits the fundamental underlying physics of molecular resonances as eigenvalues of a latent Hamiltonian matrix and is thus able to accurately describe multiple resonances simultaneously. The performance of this model is demonstrated for a range of organic molecules across chemical composition space and configuration space. We further showcase the model capabilities by predicting photoemission spectra at the level of the GW approximation for previously unseen conjugated molecules.

## Abstract 2

A physically-inspired machine learning model for orbital energies is developed that can be augmented with delta learning to obtain photoemission spectra, ionization potentials, and electron affinities with experimental accuracy.

## Graphical abstract

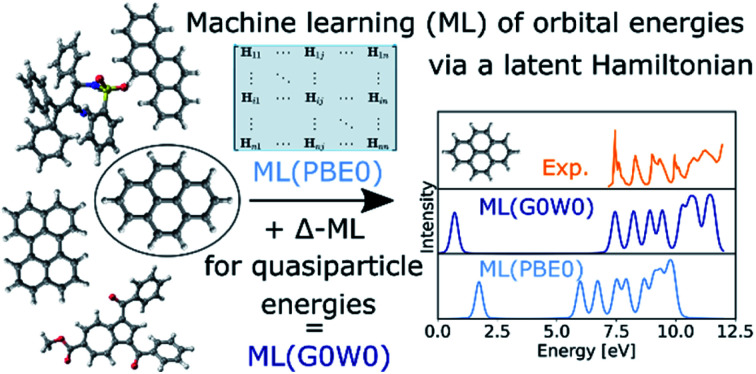

## 1 Introduction

The photoelectric effect^1^ describes the response of molecules and materials to electromagnetic radiation by emission of electrons. This effect plays a fundamental role in daily life, but also in cutting-edge technology, such as optoelectronic devices,^2,3^ regenerative electron sources for free-electron lasers,^4^ or photovoltaics, for instance to design artificial ion pumps that mimic nature.^5^

Novel functional materials in modern optoelectronic devices are often characterized by their molecular charge transport properties between acceptor and donor molecules. Such devices include organic diodes and transistors, which crucially depend on the subtle alignment of molecular acceptor and donor levels of different compounds with respect to each other. These fundamental molecular resonances associated with electron addition and removal in matter can be studied with photoemission and inverse photoemission spectroscopy.^6,7^ However, the search for optimal materials combinations is limited by the speed at which organic materials combinations can be spectroscopically characterized. This is exacerbated by the challenge of interpreting macroscopically averaged photoemission data for complex molecules.^8–11^

First-principles simulation of photoemission signatures have the potential to dramatically accelerate high throughput screening of organic materials, but the high computational cost associated with accurate many-body excited-state calculations limits their applicability to small molecular systems.^12,13^ Machine learning (ML) methods have the ability to overcome the gap between experiment and theory for spectroscopic characterization by reducing the computational effort of spectroscopic simulations without sacrificing prediction accuracy.^14,15^

ML methods in the context of spectroscopy have previously focused on predicting single energy levels,^15–19^ oscillator strengths,^20,21^ dipole moments,^22–24^ highest occupied molecular orbital (HOMO) and lowest unoccupied molecular orbital (LUMO) energies^25–28^ or band gaps.^29–31^ They have also been applied successfully to identify and characterize structures from X-ray absorption spectra.^32–34^ Electronic excitations of molecules across chemical compound space show crossings of states with different character and discontinuous behaviour. For ML models based on smooth features to capture this behaviour while simultaneously predicting multiple electronic excitations is a formidable challenge.^15,35^ By predicting spectral lineshapes^36,37^ or continuous densities-of-states^38^ directly, some of these problems can be circumvented as spectral signatures are smooth. Furthermore spectra can be represented by basis functions or discrete grids providing a consistent representation that is independent of the number of energy levels or the size of the molecule.^39–41^ However, a consequence of this simplification is that direct information on the number and character of the molecular resonances is lost.

In this work, we develop a deep convolutional neural network that accurately predicts molecular resonances across a wide range of organic molecular compounds. We encode the fundamental physics of molecular resonances by representing them *via* a Hamiltonian matrix associated with a closed set of secular equations. In contrast to previous efforts,^42–45^ this matrix representation is not based on local atomic orbital features and the elements of this matrix have no direct physical correspondence beyond the fact that the matrix eigenvalues correspond to the learned molecular resonances. As we are only training on rotationally invariant quantities, the model achieves this without the need to explicitly encode vectorial^46–49^ or tensorial equivariance properties^23,25^ beyond the rotationally invariant representation of the input molecular coordinates.^28^ The simple algebraic modification of describing vectorial targets by diagonalization of a matrix output leads to increased learning rates, reduced prediction errors, and increased transferability in predicting electron addition and removal energies across molecular composition space. We showcase the capabilities of this model by predicting photoemission spectra of previously unseen organic electronics precursor molecules at the level of Density Functional Theory (DFT). We further show that the model can be augmented to account for solvation effects or many-body electron correlation effects using only a small fraction of the original training data. Correlation effects are described at the level of GW many-body perturbation theory, which provides spectroscopic predictions of large, complex molecules in close agreement with experiment.

## 2 Results

### 2.1 Scalar, vectorial, and matrix-valued deep learning representations of molecular resonances

**Fig. 1:**
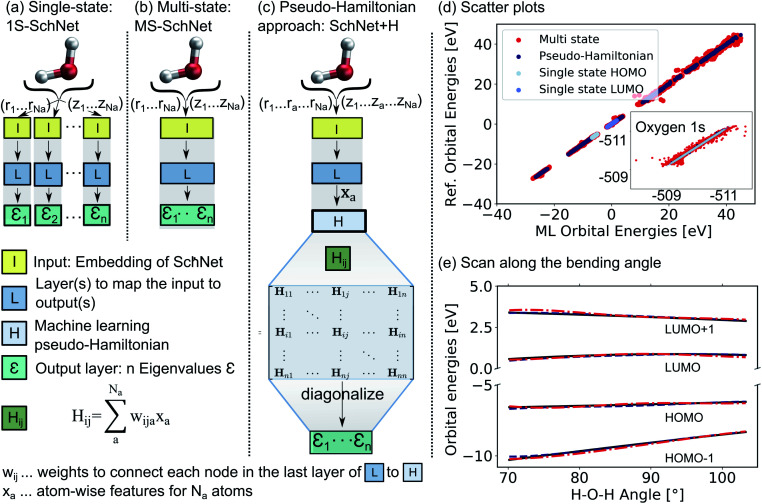
Comparison of the architecture of (a) a conventional single-state ML model (1S-SchNet), (b) a multi-state ML model (MS-SchNet), and (c) the proposed pseudo-Hamiltonian model (SchNet + H) along with the prediction accuracy for fitting 15 eigenvalues of the H_2_O molecule. The elements of the Hamiltonian matrix, *H*_ij_, are obtained by pooling atomic features, **x**_a_, from the last layer of the network **L**. (d) Scatter plots show the ML-fitted eigenvalues of a test set plotted against the reference eigenvalues. (e) Orbital energies around the HOMO–LUMO gap are plotted along the bending mode of the molecule using the MS-SchNet and SchNet + H models.

The deep convolutional neural network we propose is based on the SchNet framework^28,50^ and its architecture is illustrated in Fig. 1.

In order to learn *n* molecular resonances with the conventional scalar SchNet model, *n* ML models, one for every electronic state or resonance i need to be trained. In the following, we refer to this as a one-state (1S) model (panel a). Similarly, a vector of *n* molecular resonances can be represented using one ML model with a single vectorial output, which we refer to as multi-state (MS) model (panel b).^51^ This is identical to a previously proposed model in the context of photochemistry.^35^ The pseudo-Hamiltonian model (SchNet + H), which we propose here is shown in panel c and internally builds an ML basis that satisfies the properties of a quantum mechanical Hamiltonian, *i.e.*, it is symmetric and has eigenvalues that correspond to electron addition/removal energies. The dimension of the effective Hamiltonian output layer scales with the number of eigenvalues defined by the user. This is in contrast to a full quantum mechanical Hamiltonian, which scales with the size of the molecular system. This advantage makes it feasible to learn a large set of molecular resonances in a defined energy range for molecules of arbitrary size. The eigenvalues are obtained after diagonalization of the ML pseudo-Hamiltonian. Further details on the model training are given in the Methods Section 4.

The prediction accuracy of the three models is first analyzed by training on the 15 lowest Kohn–Sham DFT eigenvalues of 1000 configurations of the H_2_O molecule generated by *ab initio* molecular dynamics (for details on the training data, see ESI†) as shown in panels d and e of Fig. 1. As can be seen from the scatter plots in Fig. 1d and the prediction errors reported in Table S1,† the set of 15 1S models shows an accurate prediction of eigenvalues compared to the reference values with mean absolute errors (MAEs) ranging from 0.6 meV up to 5.5 meV for a given orbital energy. This is known and expected as each model only has to cover a small energy range.^28^ A single deep neural network with multi-variate outputs to predict all 15 eigenvalues shows substantial deviation between reference and prediction across all energies, *i.e.*, for low-lying semi-core as well as for valence and virtual eigenstates (panel e) with MAEs of up to 300 meV. The MS model is about twenty times less accurate in terms of MAEs of the HOMO energy than the 1S models (52 meV *vs.* 2 meV). This finding is in line with similar models reported in the literature.^17,18,22,26,27,35,39,42^

The lack of prediction accuracy of the MS model can be understood as the model has to cover a large range of energies while having to capture the dependence of each eigenvalue as a function of input. In contrast, our proposed model, SchNet + H, which learns eigenvalues indirectly *via* the pseudo Hamiltonian matrix, faithfully reproduces orbital energies across the whole energy range. The maximum MAE is 67 meV and the HOMO orbital energy can be predicted with 26 meV accuracy. Analysis of the learning behaviour shows that the prediction error decreases faster with the number of data points for the SchNet + H model compared to the MS model (see ESI Fig. S1†). In Fig. 1e, the predicted and reference eigenvalue energies of frontier orbitals around the HOMO energy are plotted as a function of the bending angle in H_2_O. While all models provide a qualitatively correct description of the smooth dependence, the MS model shows larger deviations with respect to the reference values compared to the SchNet + H model.

### 2.2 Predicting molecular resonances across chemical space

**Fig. 2:**
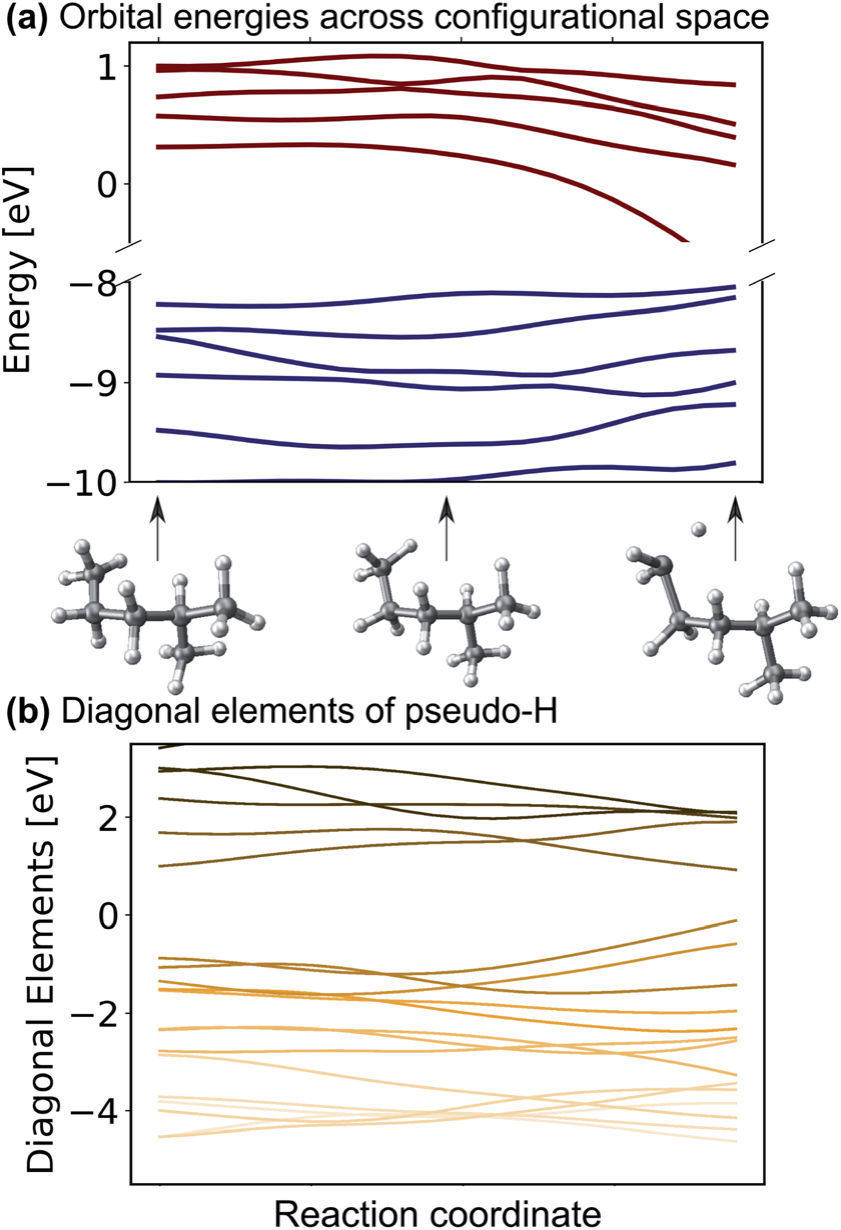
(a) Eigenvalues and (b) diagonal matrix elements of the pseudo-Hamiltonian of the SchNet + H model trained on molecules of the QM7-X data set^52^ along a trajectory of conformational change in 2-methylpentane.

One might be able to attribute the improved performance of the SchNet + H model compared to MS-SchNet simply to the increased size of the output layer which provides more flexibility. We note that both MS-SchNet and SchNet + H have almost the same number of parameters and even a further increase of the number of nodes and layers in the MS-SchNet model does not yield a better prediction (see ESI† for more details). Instead, we attribute the improved accuracy of SchNet + H to the fact that the matrix elements of the pseudo-Hamiltonian are much smoother functions in chemical space than the molecular resonances on which the model is trained. By decoupling the algebraic diagonalization that gives rise to avoided crossings and non-differential behaviour of molecular resonances from the ML model, we train an effective representation with smoother coordinate dependence. This can be seen in Fig. 2 where the orbital energies and diagonal matrix elements predicted by the SchNet + H model are shown along a reaction coordinate of 2-methylpentane. The structures are part of the first subset of the QM7-X data set^52^ on which the SchNet + H model has been trained. The QM7-X data set is an extension of QM7 (ref. 53) that contains 4.2 M equilibrium and non-equilibrium structures of a large number of molecules across chemical compound space. The quantum machine data sets^54^ are often used as a benchmark in ML studies,^28,39,55–60^ which we have also done here (plots reporting model accuracy are given in ESI Fig. S3c†). The diagonal elements of the internally formed ML basis shown in panel b vary more continuously with molecular composition than the orbital energies shown in panel a. The diagonal elements show numerous crossings along the coordinate, which is reminiscent of the behaviour of quasi-diabatic representations often used to represent multiple electronic states in computational photochemistry.^61,62^ The smooth functional form is found for different elements of the pseudo-Hamiltonian matrix and is not only true for the diagonal elements. This finding also holds for variation across chemical composition space. In ESI Fig. S3,† we show the behaviour of eigenvalues and Hamiltonian matrix elements predicted by the ML model along a coordinate of molecules with increasing number of atoms. The smooth functional behaviour of Hamiltonian matrix elements is also discernible in this case. It can be seen that the matrix elements are randomly distributed in terms of value and position in the matrix with slightly more weight on diagonal elements for larger molecules. It is noticeable that the model makes effective use of all matrix elements.

To further validate the accuracy of the model, we train it to represent 12 Kohn–Sham eigenvalues of ethanol^42,54^ along a molecular dynamics trajectory. Scatter plots are shown in ESI Fig. S2† and errors on a hold-out test set are reported in the ESI Table S2† along with other models reported in the literature for comparison. By comparing broadly across literature, we find that SchNet + H provides the same or better accuracy for the prediction of multiple resonances (between 12 and 53 across different training sets) compared to what most other models achieve for a single molecular resonance (*e.g.* the HOMO).^17,18,26,35,39,63^ The exception to this is the atomic-orbital-based SchNOrb Hamiltonian model,^42^ which predicts an average MAE for the same 12 eigenvalues of about 0.02 eV. However, we note that SchNOrb is a much larger and more flexible model, which is trained on eigenvalues and Hamiltonian matrices to predict all molecular eigenvalues (with a total averaged MAE of 0.48 eV). SchNOrb in its current form can only predict eigenvalues as a function of atomic positions for a fixed molecular composition.

**Fig. 3:**
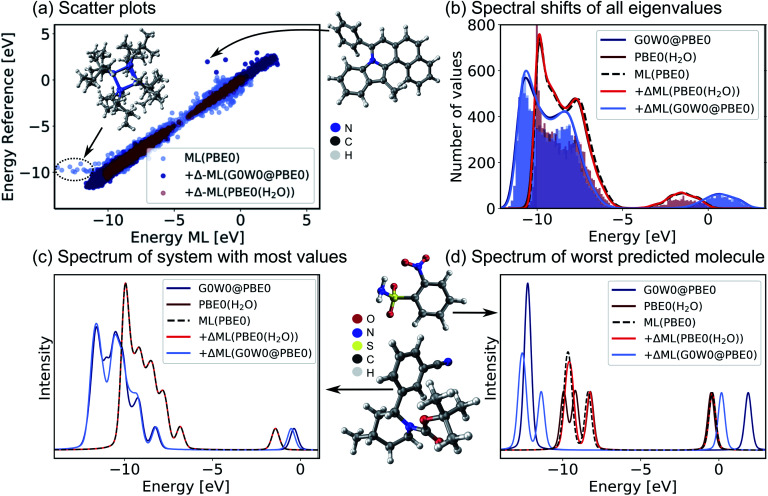
Validation of the SchNet + H model to predict PBE0 eigenvalues of the OE62 data base and the Δ-ML model that corrects the PBE0 fitted eigenvalues to G0W0@PBE0 accuracy or to PBE0 + implicit water solvation. (a) Scatter plots of a test set show the accuracy of each model. (b) Histograms of orbital eigenvalue (quasiparticle) energies for PBE0 in implicit water solvation and G_0_W_0_@PBE0 are shown for the GW5000 data set. A Gaussian envelope with 0.5 eV width is placed over each peak to depict the energy shifts between data sets and ML models. The eigenvalues of (c) the molecule with most eigenvalues within the modelled energy range and with (d) the worst predicted eigenvalues in the test set are shown using a Pseudo-Voigt lineshape^64,65^ based on a 30% Lorentzian and 70% Gaussian ratio with 0.5 eV width.

Encouraged by the promising performance of SchNet + H, we have trained a transferable model of molecular electronic states based on the OE62 data base.^66^ This data set is especially challenging as it features greater elemental diversity and more heteroatoms and functional groups than there are in the QM9 or QM7-X data bases.^26,66^ The 62k molecules in OE62 are selected from known molecular crystal structures in the Cambridge Structural Database.^67^ For each equilibrium structure, the data set reports Kohn–Sham orbital eigenvalues calculated at the PBE + vdW and hybrid PBE (PBE0) functional level of DFT. The SchNet + H model trained on the PBE0 orbital energies is termed ML(PBE0). The predicted orbital energies against reference values of a test set are shown in Fig. 3a in light blue. The SchNet + H model is trained to capture up to 53 electronic states between −10 eV up to and including the LUMO+1 state. The model error for each data point in the whole training set shows a very large deviation for some systems with particularly high structural complexity. One such outlier is shown in panel a, which contains an 8-membered nitrogen cage in the center (see also Fig. S4 in the ESI†). We note that these data points do not influence the model accuracy and its ability to generalize across chemical compound space, which we have tested by removing outliers and retraining the model. Training errors are further reported along with the number of training data in ESI Table S2.† The model error (MAE of 0.13 eV) is quite convincing with few prominent deviations at low orbital energies that are associated with a small number of outlier molecules of particularly high structural complexity.

For a subset of 30 876 molecules, the OE62 set further reports PBE0 (ref. 68) eigenvalues calculated with the Multipole Expansion (MPE) implicit solvation method.^69^ For a further subset of 5239 molecules in vacuum (termed GW5000), the data set reports quasiparticle energies calculated at the many-body perturbation theory in the G_0_W_0_@PBE0 approximation.^70–72^ With the exception of the HOMO, Kohn–Sham orbital energies lack a physical meaning^73^ and important properties of optoelectronic materials, such as donor and acceptor levels^20,39^ or band gaps are often incorrectly described.^70^ In order to obtain charged excitations in molecules and materials, the GW method^13,71^ can be used to correct artifacts that arise from approximations in the exchange–correlation functional in DFT. The computation of quasiparticle energies is computationally unfeasible for the full OE62 data set and for much larger molecular systems with potential relevance in organic electronics. The electronic resonances that include solvation effects and correlation effects captured in the two data subsets should principally deviate from the PBE0 energies of the full data set in relatively systematic ways. We therefore apply a Δ-ML approach^20,74^ to train ML models to capture the difference in orbital energy and quasiparticle energy between PBE0 in vacuum and in water and PBE0 and G_0_W_0_@PBE0, respectively. Our Δ-ML approach is explained in more detail in the Methods section. Briefly, the SchNet + H model of the PBE0 eigenvalues learns a baseline for the full 62k data set (50k training data points), whereas the Δ-ML models learn the difference with respect to this ML(PBE0) baseline from a much smaller training data set (4k).

Test errors of orbital (quasiparticle) energies predicted by the two Δ-ML models are also reported in Fig. 3a. We note that the error distribution is narrower for the Δ-ML-corrected models than for ML(PBE0). Fig. 3b shows that the ML(PBE0) and the two Δ-ML models predict eigenenergies with high fidelity and accurately represent the data sets with a MAE (RMSE) as low as 2 and 4 meV for PBE0(H2O) and G0W0@PBE0, respectively. On closer inspection, we find that the excitation spectrum of the molecule in the test set with the most eigenvalues in the represented energy range shows quantitative agreement with the reference spectrum and a MAE (RMSE) of 29 (52) meV in the vicinity of the peaks (see Fig. 3c). The spectrum for the molecule with the highest prediction error (Fig. 3d) shows noticeable deviations only for the Δ-ML(G0W0@PBE0) model. Here the model predicts a splitting of the HOMO levels and underestimates the energy of the LUMO compared to the reference data with a MAE of 0.51 meV and a RMSE of 0.94 meV on the spectrum in the vicinity of the peaks. We note that this molecule is a rare case in the data base that contains more heteroatoms than carbon atoms, which could be a reason for the increased prediction errors.

The Δ-ML(G0W0@PBE0) is only trained on a subset of 4k datapoints of the GW5000 data set as no quasiparticle energies are available for the full 62k data points of the OE62 data set. By applying the SchNet + H ML(PBE0) and Δ-ML(G0W0@PBE0) models to predict the quasiparticle energies of the full OE62 data set, we can gauge the transferability of the models across chemical space. We find that the models predict the same vertical shift of occupied and unoccupied states between PBE0 and G0W0@PBE0 levels of theory for the full OE62 data set that we have shown in Fig. 3b for the GW5000 set (see ESI Fig. S4b†). In addition, the predictions show a linear correlation of the Kohn–Sham HOMO and LUMO orbital energies with the corresponding quasiparticle energies (Fig. S4a†). This linear relation has previously been identified for HOMO energies of the smaller GW5000 subset in ref. 66, which we can now extend for all orbitals in the OE62 set. Not surprisingly, the application of the Δ-ML(G0W0@PBE0) induces a downward shift of occupied PBE0 energies and an upward shift in energy for unoccupied orbitals to create electron removal and addition quasiparticle energies. Hardly any shift can be found for the eigenenergies obtained from the implicit solvation model indicating that solvation has a minor impact on the molecular resonances.

The combined SchNet + H ML(PBE0) and Δ-ML(G0W0@PBE0) models can predict (inverse) photoemission spectra, ionization potentials and electron affinities of large and complex organic molecules which are well out of reach for *ab initio* calculations at this level of theory. Previous works have predicted individual HOMO and LUMO quasiparticle energies of the GW5000 (ref. 27) and GW100 (ref. 63 and 78) data sets. Our model is able to predict many quasiparticle resonances over a wide energy range and is therefore able to simulate photoemission spectra.

### 2.3 Prediction of energy levels and photoemission spectra of functional organic molecules

In the following, we report the ML-based prediction of the photoemission spectra of a range of organic molecules which are commonly used as acceptor and donor compounds in organic electronics applications. To showcase the wide applicability of our model, three different types of functional organic molecules are selected: azenes, derivatives of azulenes, and other polycyclic aromatic hydrocarbons. Azulenes are particularly interesting as they exhibit unusually low HOMO–LUMO gaps for molecules of such small conjugation length due to their topological properties.^79,80^ Polycyclic aromatic hydrocarbons are often considered for the design of new organic light-emitting diode materials, field-effect transistors or photovoltaics.^3,7,81^ Their electronic properties make these molecules not only relevant for optoelectronic applications, but also for other research areas such as astrochemistry^82^ and atmospherical chemistry.^83^

**Fig. 4:**
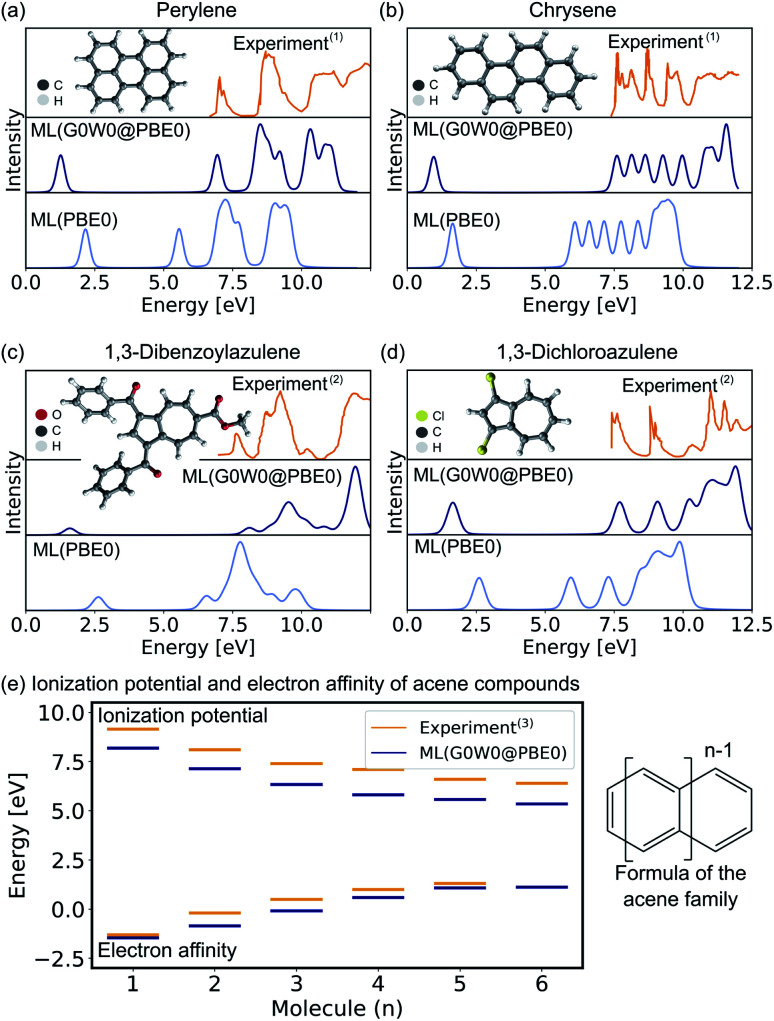
Experimental and ML predicted photoemission spectra along with the LUMO (quasiparticle) orbital energies at the PBE0 (G0W0@PBE0) level for (a) perylene, (b) chrysene, (c) 1,3-dibenzoylazulene, and (d) 1,3-dichloroazulene. A Pseudo-Voigt lineshape^64,65^ based on a 30% Lorentzian and 70% Gaussian ratio with 0.3 eV width was used. (e) Electron affinities and ionization potentials of acene molecules are plotted with increasing ring size. ^(1)^Experimental photoemission spectra have been extracted from ref. 75, ^(2)^ ref. 76, and ^(13)^ ref. 77.

The excitation spectra are predicted with the ML model trained on PBE0 orbital energies of the OE62 data set (denoted as ML(PBE0)) and the Δ-ML model trained on the difference of the ML(PBE0) model and the G0W0@PBE0 values of 4k datapoints of the GW5000 data set. The combination of both models is denoted as ML(G0W0@PBE0) in the following. All photoemission spectra shown in Fig. 4a–d and ESI Fig. S6–S8† are ML predictions of molecules the model has not seen before. In addition to the photoemission spectra, the LUMO energies are plotted and the spectra obtained from Kohn–Sham eigenvalues are shown to highlight the Δ-ML quasiparticle correction. The spectra obtained with ML(G0W0@PBE0) are in excellent agreement with experiment. Compared to spectra based on Kohn–Sham orbital energies, they accurately reflect the positions and intensities of photoemission features. In addition, the model correctly predicts the spectral fingerprints of similar molecules and accurately describes substituent effects. For instance, the model accurately predicts the differences of 1,3-dibromoaculene and 1,3-dichloroaculene (see panel d and ESI† for details). Even a highly complex molecule such as 1,3-dibenzoylazulene with 48 atoms (see Fig. 4d), is predicted with high accuracy with respect to the experimental spectrum.

In addition to the photoemission spectra, we predict the electron affinities and ionization potentials of molecules of the acene family. As can be seen in Fig. 4d, acenes are built from linearly condensed benzene rings and are often referred to as “1d graphene strips”. Acenes are especially interesting as they are relevant in electronic devices due to their narrow HOMO–LUMO gaps that can result in generally high conductivity.^2,77^ The predicted ionization potentials and electron affinities fit well to experimental values although the HOMO–LUMO gaps are slightly underestimated. This underestimation is not an artifact of the ML model, but is a well known limitation of the G0W0 method for acene molecules.^77^ Due to the instability of hexacene (*n* = 6), the experimental prediction of charged excitations is challenging, hence no electron affinity value is available to which the ML predictions can be compared.^2^ The respective photoemission spectra are reported in ESI Fig. S8† and are in qualitatively good agreement with experimental spectra reported in literature.^77^

## 3 Conclusion

In this work, we have developed a machine learning model that can be used to predict orbital energies of large and complex molecules in various configurations during molecular dynamics and orbital and quasiparticle energies across chemical compound space in general. By using physical relations and building an internal ML basis that exploits the fundamental symmetries of a quantum chemical Hamiltonian, but does not scale with system size, molecular resonances such as orbital and quasiparticle energies can be predicted with high accuracy. The developed model is accurate enough to be used in combination with a Δ-ML model trained on the difference between the ML predicted orbital energies of DFT and quasiparticle energies from many-body perturbation theory. This provides an extremely data-efficient way to eliminate errors in spectral signatures that arise from exchange–correlation approximations in Kohn–Sham DFT and to achieve close to experimental accuracy in the prediction of photoemission spectra, ionization potentials, and electron affinities. We evidence this by predicting these quantities with high accuracy compared to experiment for unseen azulene-like molecules, acenes, and polyaromatic hydrocarbons that are often targeted for the design of new organic electronic materials.^3^ The model clearly has the ability to distinguish between functional groups and predict trends as a function of molecule size in conjugated systems. The results demonstrate the transferability and scalability of the model. While we have only shown the application of this model for frontier orbital and quasiparticle energies, we are confident that it will be similarly applicable to the prediction of core-levels and X-ray photoemission signatures.^6,41^

The ability to efficiently predict molecular resonances at high accuracy is key to enable large-scale computational screening of novel acceptor and donor molecules to be used in organic electronics and thin film device applications.^7,81,84^ We expect that the presented method will be very useful in this context. It will likely be especially powerful in combination with generative ML^85,86^ or reinforcement learning models^87^ that can recommend new molecular structures with specific tailored properties. In this way, a fully automated search algorithm for new molecules with optimally tuned acceptor and donor levels could be created.^81,88,89^

## 4 Methods

The underlying ML model used in this work is SchNet.^28,90^ As the network architecture of SchNet is explained in the original references in details, we will only briefly describe it here: SchNet is a convolutional message-passing neural network that was originally developed to model scalar valued properties and their derivatives^91^ and has recently been extended to model multiple energy levels and multi-state properties in the context of molecular excited states. This model was previously termed SchNarc and we call it MS-SchNet for consistency in this work.^35,92^

### 4.1 SchNet + H

(MS-)SchNet combines a network that learns the molecular representation in an end-to-end fashion with a network that maps this tailored representation to the targeted outputs. The first part of the network, the input layer **I** in Fig. 1, takes atomic positions, *r*_1_ to *r*_Na_, with *N*_a_ being the number of atoms in a system, and elemental charges, *z*_1_ to *z*_Na_, as an input. It transforms this information into atomistic descriptors using filter-generating networks and atom-wise layers to optimize the representation. This representation enters into the network, **L** in Fig. 1, which itself contains layers that learn atomistic features *x*_a_. These features are sum-pooled and usually form (excitation) energies. The SchNet + H model developed here is an adaption of MS-SchNet, in which the architecture of the network is altered such that the final fully-connected layer represents a symmetric matrix, **H**^ML^ (*H* in Fig. 1), that returns a diagonal matrix of *n* eigenvalues *ε*^ML^_i_ after diagonalization:

1 diag({*ε*^ML^_i_}) = **U**^T^**H**^ML^**U**.

As SchNet learns the molecular representation, the need for extensive hyperparameter search is reduced. As illustrated in Fig. 1, Hamiltonian elements for states i and j, *H*_ij_, are obtained by sum-pooling of atomic features, **x**_a_. *w*_ija_ denotes the weights that connect the last layer of the standard SchNet network to the pseudo-Hamiltonian layer.2
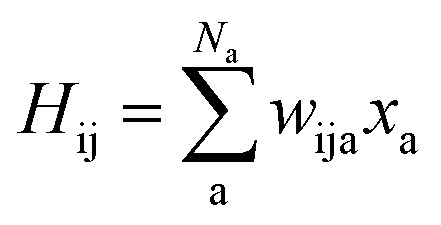


Diagonalization of the pseudo-Hamiltonian matrix is carried out after each pass trough the network and the eigenvalues predicted by the ML model enter the loss function, *L*_2_:3
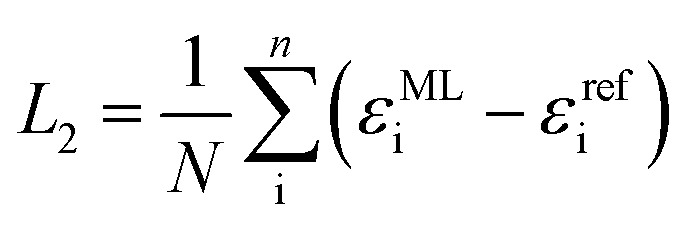
where *ε*^ref^_i_ indicate reference eigenvalues in the training data set. Due to the fact that we backpropagate through the diagonalization, the atom-wise features are connected and form a global molecular representation of the orbital energies.

SchnNet + H models consistently provide better accuracy than MS-SchNet models. While the accuracy of direct training in MS-SchNet can be improved by placing a Gaussian function on top of the orbital energies in the loss function, this did not lead to more accurate results than the SchNet + H model. Our goal was to develop a model that predicts molecular resonances across chemical space and does not scale with system size. We therefore define an energy range within which we represent all orbital energies up to a maximum number of values that defines the size of **H**^ML^. The energy range that was fitted for each data set is reported in ESI Table S2.† A varying number of orbital energies are used for training with the maximum number of eigenvalues being 53 for the OE62 and GW5000 training sets.^66^ Every molecule that contains fewer orbital energies than the maximum amount of fitted values can be predicted by using a mask in the loss function that makes sure only relevant values are included.

### 4.2 Δ-MS-SchNet

The GW5000 training set contains 5k data points and represents a subset of the OE62 data set with G0W0@PBE0 quasiparticle energies. Due to the complexity of the data set with molecules up to 100s of atoms, 5k data points are not enough to train a model directly on quasiparticle energies (MAEs of 0.3 eV). To circumvent this problem, Δ-ML^20^ was applied. This approach can be used to train the difference between a baseline method and a higher accuracy method. In this case, we trained a model on the difference between the orbital energies obtained from DFT as predicted by the SchNet + H model, *ε*^ML^(DFT), and the quasiparticle energies of G0W0@PBE0, *ε*^QC^(G0W0):

4 Δ*ε*^ML^(G0W0 − DFT) = *ε*^ref^(G0W0) − *ε*^ML^(DFT)

For the Δ-ML model, a conventional MS model is sufficient as the differences in DFT (predicted by the SchNet + H model) and G0W0 vary less strongly as a function of input than the actual targets.^93,94^ The architecture of the Δ-ML model is identical to panel (b) in Fig. 1. The Δ-ML model is trained separately from the SchNet + H model and is not combined in an end-to-end fashion. Nevertheless, the models depend on each other as the SchNet + H models provides the baseline for the Δ-ML model and predictions of both models need to be combined to obtain reliable quasiparticle energies.

Although the accuracy of the Δ-models can be improved by using DFT reference values as the baseline for Δ-models (MAE of 0.02 eV are obtained with DFT baseline models compared to MAEs of 0.16 eV with SchNet + H(PBE0) baseline models), the ML predicted DFT values are chosen as a baseline to circumvent the use of DFT reference calculations for new predictions altogether. This provides an ML prediction that is independent of electronic structure calculations and practical for large-scale screening studies. The predicted G0W0@PBE0 values are obtained by using the following equation:

5 *ε*^ML^(G0W0) = *ε*^ML^(DFT) + Δ*ε*^ML^(G0W0 − DFT).

For the prediction of G0W0@PBE0 values, we thus use two ML models, one SchNet + H model trained on DFT orbital energies and one MS-SchNet model trained on the difference between quasiparticle and orbital energies. Further details on model size, training and test set split, and model parameters can be found in the ESI.† The chosen model parameters are reported in ESI Table S3.†

### 4.3 Spectra predictions

The comparison to experimental photoemission spectra shown in Fig. 4 and ESI Fig. S5–S7† is obtained by convolution of the orbital energies to account for electronic lifetime broadening, instrument response, and many-body effects, such as inelastic losses. For the broadening we use a Pseudo-Voigt lineshape^64,65^ with 30% Lorentzian and 70% Gaussian and varying widths of 0.3–0.5 eV. The spectral shifts of all eigenvalues of molecules across chemical compound space given in Fig. 3 and ESI Fig. S4 and S7† are obtained by Gaussian convolution with a width of 0.5 eV and subsequent summation.

## Author contributions

R. J. M. proposed and supervised the project. J. W. designed and implemented the model. J. W. performed the model training, data acquisition, and analysis. J. W. and R. J. M. discussed and interpreted the data and wrote the manuscript.

## Data availability

The extracted experimental data and the data shown in the figures are available on figshare at DOI: 10.6084/m9.figshare.14212595. All code developed in this work is available on http://www.github.com/schnarc. The QM9 data were provided by Adam McSloy and will be published along with the relevant publication for which they were generated.

## Conflicts of interest

There are no conflicts to declare.

## Supplementary Material

SC-012-D1SC01542G-s001

## References

[cit1] Pendry J. (1979). Nature.

[cit2] Watanabe M., Chang Y. J., Liu S.-W., Chao T.-H., Goto K., Islam M. M., Yuan C.-H., Tao Y.-T., Shinmyozu T., Chow T. J. (2012). Nat. Chem..

[cit3] Okamoto H., in Organic Chemistry of π-Conjugated Polycyclic Aromatic Hydrocarbons: Acenes and Phenacenes, ed. Y. Kubozono, Springer Singapore, Singapore, 2019, pp. 211–228

[cit4] Liu F. (2021). et al.. Nat. Commun..

[cit5] Xiao K., Chen L., Chen R., Heil T., Lemus S. D. C., Fan F., Jiang L., Antonietti M. (2019). Nat. Commun..

[cit6] Klein B., Hall S., Maurer R. (2021). J. Phys.: Condens. Matter.

[cit7] Ishii H., Sugiyama K., Ito E., Seki K. (1999). Adv. Mater..

[cit8] Hofmann O., Zojer E., Hörmann L., Jeindl A., Maurer R. (2021). Phys. Chem. Chem. Phys..

[cit9] Norman P., Dreuw A. (2018). Chem. Rev..

[cit10] Zhan C.-G., Nichols J. A., Dixon D. A. (2003). J. Phys. Chem. A.

[cit11] Puschnig P., Reinisch E.-M., Ules T., Koller G., Soubatch S., Ostler M., Romaner L., Tautz F. S., Ambrosch-Draxl C., Ramsey M. G. (2011). Phys. Rev. B: Condens. Matter Mater. Phys..

[cit12] Quantum Chemistry and Dynamics of Excited States: Methods and Applications, ed. L. González and R. Lindh, John Wiley & Sons, 2020

[cit13] Reining L. (2018). Wiley Interdiscip. Rev. Comput. Mol. Sci..

[cit14] Westermayr J., Gastegger M., Schütt K. T., Maurer R. J. (2021). J. Chem. Phys..

[cit15] Westermayr J., Marquetand P. (2020). Chem. Rev..

[cit16] Behler J. (2017). Angew. Chem., Int. Ed..

[cit17] Zubatyuk T., Nebgen B., Lubbers N., Smith J. S., Zubatyuk R., Zhou G., Koh C., Barros K., Isayev O. and Tretiak S., arXiv:1909.12963, 2019

[cit18] Westermayr J., Gastegger M., Menger M. F. S. J., Mai S., González L., Marquetand P. (2019). Chem. Sci..

[cit19] Pronobis W., Schütt K. R., Tkatchenko A., Müller K.-R. (2018). Eur. Phys. J. B.

[cit20] Ramakrishnan R., Hartmann M., Tapavicza E., von Lilienfeld O. A. (2015). J. Chem. Phys..

[cit21] Xue B.-X., Barbatti M., Dral P. O. (2020). J. Phys. Chem. A.

[cit22] Westermayr J., Marquetand P. (2020). J. Chem. Phys..

[cit23] Zhang Y., Ye S., Zhang J., Hu C., Jiang J., Jiang B. (2020). J. Phys. Chem. B.

[cit24] Gastegger M., Behler J., Marquetand P. (2017). Chem. Sci..

[cit25] Schütt K. T., Unke O. T. and Gastegger M., arXiv:2102.03150, 2021

[cit26] Stuke A., Todorović M., Rupp M., Kunkel C., Ghosh K., Himanen L., Rinke P. (2019). J. Chem. Phys..

[cit27] Tirimbó G., Caylak O. and Baumeier B., arXiv:2012.01787, 2020

[cit28] Schütt K. T., Sauceda H. E., Kindermans P.-J., Tkatchenko A., Müller K.-R. (2018). J. Chem. Phys..

[cit29] Zhuo Y., Mansouri Tehrani A., Brgoch J. (2018). J. Phys. Chem. Lett..

[cit30] Pilania G., Gubernatis J., Lookman T. (2017). Comput. Mater. Sci..

[cit31] Isayev O., Oses c., Toher c., Gossett E., Curtarolo S., Tropsha A. (2017). Nat. Commun..

[cit32] Zheng C., Mathew K., Chen C. (2018). npj Comput. Mater..

[cit33] Timoshenko J., Lu D., Lin Y., Frenkel A. I. (2017). J. Phys. Chem. Lett..

[cit34] Timoshenko J., Anspoks A., Cintins A., Kuzmin A., Purans J., Frenkel A. I. (2018). Phys. Rev. Lett..

[cit35] Westermayr J., Gastegger M., Marquetand P. (2020). J. Phys. Chem. Lett..

[cit36] Kananenka A. A., Yao K., Corcelli S. A., Skinner J. L. (2019). J. Chem. Theory Comput..

[cit37] Sanchez-Gonzalez A. (2017). et al.. Nat. Commun..

[cit38] Fung V., Hu G., Ganesh P., Sumpter B. G. (2021). Nat. Commun..

[cit39] Ghosh K., Stuke A., Todorović M., Jørgensen P. B., Schmidt M. N., Vehtari A., Rinke P. (2019). Adv. Sci..

[cit40] Ben Mahmoud C., Anelli A., Csányi G., Ceriotti M. (2020). Phys. Rev. B: Condens. Matter Mater. Phys..

[cit41] Rankine C. D., Madkhali M. M. M., Penfold T. J. (2020). J. Phys. Chem. A.

[cit42] Schütt K., Gastegger M., Tkatchenko A., Müller K.-R., Maurer R. J. (2019). Nat. Commun..

[cit43] Qiao Z., Welborn M., Anandkumar A., Manby F. R., Miller T. F. (2020). J. Chem. Phys..

[cit44] Welborn M., Cheng L., Miller T. F. (2018). J. Chem. Theory Comput..

[cit45] Cheng L., Welborn M., Christensen A. S., Miller T. F. (2019). J. Chem. Phys..

[cit46] Chmiela S., Sauceda H. E., Poltavsky I., Müller K.-R., Tkatchenko A. (2019). Comput. Phys. Commun..

[cit47] Batzner S., Smidt T. E., Sun L., Mailoa J. P., Kornbluth M., Molinari N. and Kozinsky B., arxiv:2101.03164, 2021 10.1038/s41467-022-29939-5 PMC9068614 35508450

[cit48] Miller B. K., Geiger M., Smidt T. E. and Noé F., arXiv:2008.08461, 2020

[cit49] Thomas N., Smidt T., Kearnes S., Yang L., Li L., Kohlhoff K. and Riley P., arXiv:1802.08219, 2018

[cit50] Schütt K. T., Glawe H., Brockherde F., Sanna A., Müller K.-R., Gross E. K. (2014). Phys. Rev. B: Condens. Matter Mater. Phys..

[cit51] Westermayr J., Faber F. A., Christensen A. S., von Lilienfeld O. A., Marquetand P. (2020). Mach. Learn.: Sci. Technol..

[cit52] Hoja J., Sandonas L. M., Ernst B. G., Vazquez-Mayagoitia A., DiStasio Jr R. A., Tkatchenko A. (2021). Sci. Data.

[cit53] Rupp M., Tkatchenko A., Müller K.-R., von Lilienfeld O. A. (2012). Phys. Rev. Lett..

[cit54] http://quantum-machine.org/datasets/.

[cit55] Christensen A. S., Faber F. A., von Lilienfeld O. A. (2019). J. Chem. Phys..

[cit56] Chmiela S., Tkatchenko A., Sauceda H. E., Poltavsky I., Schütt K. T., Müller K.-R. (2017). Sci. Adv..

[cit57] Gastegger M., Schwiedrzik L., Bittermann M., Berzsenyi F., Marquetand P. (2018). J. Chem. Phys..

[cit58] Christensen A. S., Bratholm L. A., Faber F. A., Anatole von Lilienfeld O. (2020). J. Chem. Phys..

[cit59] Kim H., Park J., Choi S. (2019). Sci. Data.

[cit60] Veit M., Wilkins D. M., Yang Y., DiStasio R. A., Ceriotti M. (2020). J. Chem. Phys..

[cit61] Köppel H., Gronki J., Mahapatra S. (2001). J. Chem. Phys..

[cit62] Shu Y., Truhlar D. G. (2020). J. Chem. Theory Comput..

[cit63] Rahaman O., Gagliardi A. (2020). J. Chem. Inf. Model..

[cit64] Schmid M., Steinrück H.-P., Gottfried J. M. (2014). Surf. Interface Anal..

[cit65] Schmid M., Steinrück H.-P., Gottfried J. M. (2015). Surf. Interface Anal..

[cit66] Stuke A., Kunkel C., Golze D., Todorović M., Margraf J. T., Reuter K., Rinke P., Oberhofer H. (2020). Sci. Data.

[cit67] Allen F. H. (2002). Acta Crystallogr. B.

[cit68] Adamo C., Barone V. (1999). J. Chem. Phys..

[cit69] Sinstein M., Scheurer C., Matera S., Blum V., Reuter K., Oberhofer H. (2017). J. Chem. Theory Comput..

[cit70] Golze D., Dvorak M., Rinke P. (2019). Front. Chem..

[cit71] Hedin L. (1965). Phys. Rev..

[cit72] Aryasetiawan F., Gunnarsson O. (1998). Rep. Prog. Phys..

[cit73] Stowasser R., Hoffmann R. (1999). J. Am. Chem. Soc..

[cit74] Bogojeski M., Vogt-Maranto L., Tuckerman M., Müller K.-R., Burke K. (2020). Nat. Commun..

[cit75] Dougherty D., Lewis J., Nauman R., McGlynn S. (1980). J. Electron Spectrosc. Relat. Phenom..

[cit76] Deleuze M. S. (2002). J. Chem. Phys..

[cit77] Rangel T., Berland K., Sharifzadeh S., Brown-Altvater F., Lee K., Hyldgaard P., Kronik L., Neaton J. B. (2016). Phys. Rev. B: Condens. Matter Mater. Phys..

[cit78] van Setten M. J., Caruso F., Sharifzadeh S., Ren X., Scheffler M., Liu F., Lischner J., Lin L., Deslippe J. R., Louie S. G., Yang C., Weigend F., Neaton J. B., Evers F., Rinke P. (2015). J. Chem. Theory Comput..

[cit79] Xin H., Gao X. (2017). ChemPlusChem.

[cit80] Chen Y., Zhu Y., Yang D., Zhao S., Zhang L., Yang L., Wu J., Huang Y., Xu Z., Lu Z. (2016). Chem.–Eur. J..

[cit81] Yamaguchi Y., Takubo M., Ogawa K., Nakayama K.-i., Koganezawa T., Katagiri H. (2016). J. Am. Chem. Soc..

[cit82] Lemmens A. K., Rap D. B., Thunnissen J. M., Willemsen B., Rijs A. M. (2020). Nat. Commun..

[cit83] Cachada A., Pato P., Rocha-Santos T., da Silva E. F., Duarte A. (2012). Sci. Total Environ..

[cit84] Niskanen J., Sahle C. J., Gilmore K., Uhlig F., Smiatek J., Föhlisch A. (2017). Phys. Rev. E.

[cit85] Gebauer N., Gastegger M., Schütt K. (2019). Adv. Neural Inf. Process. Syst..

[cit86] Mercado R., Rastemo T., Lindelöf E., Klambauer G., Engkvist O., Chen H., Bjerrum E. J. (2020). Mach. Learn.: Sci. Technol..

[cit87] Simm G., Pinsler R. and Hernandez-Lobato J. M., Proceedings of the 37th International Conference on Machine Learning, 2020, pp. 8959–8969

[cit88] Peng S.-P., Zhao Y. (2019). J. Chem. Inf. Model..

[cit89] Elton D. C., Boukouvalas Z., Fuge M. D., Chung P. W. (2019). Mol. Syst. Des. Eng..

[cit90] Schütt K. T., Kessel P., Gastegger M., Nicoli K. A., Tkatchenko A., Müller K.-R. (2019). J. Chem. Theory Comput..

[cit91] Schütt K. T., Kindermans P. J., Sauceda H. E., Chmiela S., Tkatchenko A. and Müller K. R., Advances in Neural Information Processing Systems, 2017, pp. 992–1002

[cit92] SchNarc, https://github.com/schnarc/SchNarc

[cit93] Raghunathan Ramakrishnan M. R., Dral P. O., von Lilienfeld O. A. (2014). Sci. Data.

[cit94] Dral P. O., Owens A., Dral A., Csányi G. (2020). J. Chem. Phys..

