# Supplementary material for: Physically inspired deep learning of molecular excitations and photoemission spectra
Source: Chem Sci. 2021 Jun 30;12(32):10755–64. doi: 10.1039/d1sc01542g (PMC8372319; doi:10.1039/d1sc01542g)
Supplement: SC-012-D1SC01542G-s001 [file SC-012-D1SC01542G-s001.pdf]

Electronic Supplementary Material (ESI) for Chemical Science.
This journal is © The Royal Society of Chemistry 2021

Supporting Information for "Physically inspired deep learning of molecular excitations and photoemission spectra"

Julia Westermayr and Reinhard J. Maurer[a)]

Department of Chemistry, University of Warwick, Gibbet Hill Road, Coventry, CV4 7AL, United Kingdom

CONTENTS

S1. Training 2

A. Training sets 2

B. Model parameters 2

S2. Training on 15 eigenvalues of water 3

S3. Performance of ML models on eigenvalues of ethanol, QM7-X, QM9, OE62 and GW5000 molecules 5

S4. Spectra prediction of unseen molecules in addition to the main text 10

References 12

[a)]Electronic mail: r.maurer@warwick.ac.uk

S1. TRAINING

A. Training sets

The training sets for the water and ethanol molecules are taken from Ref. 1. The training set for water and ethanol contained 5k and 30k data points, respectively, at PBE[2]/def2-SVP level of theory. The Hamiltonian matrices and the overlap matrices for the QM9 data set[3,4] are obtained from authors of Ref. 5 and are computed at the same level of theory using a final grid of 5, very tight SCF convergence criteria and the program ORCA.[6] The Hamiltonian and overlap matrices are used to generate the eigenvalues that are subsequently learned. The orbital energies for the QM7-X data set are directly obtained from Ref. 7 and the OE62 as well as the GW5000 data set were obtained from Ref. 8, both reporting orbital energies for a diverse set of molecules at PBE0 level of theory.[9] The GW5000 data set further contains orbital energies at G0W0@PBE0 that are computed in accordance to the GW100 benchmark set[10] and PBE0 level of theory including implicit solvation (PBE0(H_2O)).[11] For the H_2O molecule, the 15 energetically lowest eigenvalues are fitted. For ethanol, 12 eigenvalues between an energy range of -54 eV and +3 eV are fitted. With respect to the QM9 data set, we fit 34 eigenvalues within an energy range of -54 eV and +1 eV. To allow for better comparison of a multi-state (MS) ML model reported in Ref. 12, the highest occupied 16 molecular orbital energies of the QM9 data set are additionally fitted. For the QM7-X data set, an energy range of -54 eV to 1 eV was used and an energy range of -10 eV to the LUMO+1 (LUMO) orbitals is used for the OE62 (GW5000) data set, resulting in a maximum number of 30 eigenvalues for the molecules in the QM7-X data set and a maximum number of 53 (52) orbital (quasiparticle) energies for molecules in the OE62 (GW5000) data set. In order to allow for fitting of a very diverse range of molecules we do not discard any molecules for training that contain less than the maximum number of orbitals in a molecule within the defined energy range, but neglect those parts of the eigenvalue vector that contain values outside the defined energy range when optimizing the fitting parameters of the model.

B. Model parameters

The model parameters are optimized by splitting each data set into training, validation, and test set using random splits. The validation set is used to avoid overfitting and for validation. The final model accuracy is reported on the test set in Table S1 and S2. As our model uses the SchNet descriptor, two networks function end-to-end. Thus, the cutoff, the interaction layers, the number of features, and the number of Gaussian functions to represent the molecule and to learn an optimal representation have to be optimized in addition to the number of hidden layers, nodes per hidden layer, the learning rate, and the batch size. The model hyperparameters were optimized on a random grid.

Unless stated otherwise, a batch size between 16 and 32 and default MS-SchNet[13,14] parameters with a cutoff of 5 or 6 Bohr are used. Lower and upper limits for the interaction layers, hidden layers for mapping the representation to the pseudo-Hamiltonian layer, Gaussian functions, features, nodes per hidden layer, and the learning rate were 3-6, 3-6, 25-100, 128-1024, 100-1500, and 0.001-0.01, respectively. Different from default parameters, 25 Gaussian functions are used instead of 50. Based on the training set size, the learning rate is varied between 0.001 and 0.0001 with larger values for smaller training sets. In case of ethanol, the QM7-X, QM9, and GW5000 data sets, we use 512, 512, 1024, and 512 features,

respectively. For the QM9, OE62, and GW5000 data set, 4 layers are used instead of 3. The number of nodes is increased to 500 for the MS-SchNet model to fit GW5000 Δ-values.

S2. TRAINING ON 15 EIGENVALUES OF WATER

For the 15 eigenvalues of H_2O we train 15 single-state (1S-SchNet) models, one multi-state (MS-SchNet) model and one pseudo-Hamiltonian model (SchNet+H). The mean absolute errors (MAEs) and root-mean squared errors (RMSEs) for every energy level are reported in Table S1 in addition to Fig. 1 d and e in the main text.

Eigenvalue	1S-SchNet	MS-SchNet	SchNet+H
HOMO-4	0.6 (3.0)	54.6 (79.1)	14.9 (24.1)
HOMO-3	0.7 (5.8)	51.4 (75.4)	45.9 (32.7)
HOMO-2	1.1 (2.8)	50.8 (90.3)	21.8 (42.4)
HOMO-1	3.4 (12.4)	50.0 (78.3)	23.0 (36.7)
HOMO	2.0 (4.0)	51.7 (75.2)	25.6 (41.6)
LUMO	0.8 (2.1)	59.1 (84.1)	26.7 (35.6)
LUMO+1	0.6 (1.5)	51.4 (85.1)	22.4 (107)
LUMO+2	5.5 (31.1)	136 (228)	66.5 (90.1)
LUMO+3	5.3 (15.4)	144 (231)	59.7 (46.7)
LUMO+4	1.1 (4.2)	40.2 (66.2)	25.7 (50.3)
LUMO+5	5.0 (7.9)	52.8 (78.3)	32.9 (61.5)
LUMO+6	2.4 (8.2)	90.4 (133)	34.6 (84.5)
LUMO+7	2.5 (8.7)	202 (298)	60.6 (47.1)
LUMO+8	2.7 (8.9)	297 (420)	36.9 (180)
LUMO+9	2.9 (9.5)	25.6 (297)	51.7 (115)

TABLE S1. Mean absolute (root mean-squared) errors in meV of the different orbitals predicted with 15 single-state models, a multi-state model, and the pseudo-Hamiltonian model.

Moreover, we compute the learning curves for the MS and SchNet+H models using a network architecture with comparable number of fitting parameters, i.e., 369871 and 373336 parameters, respectively. We further test larger MS models, containing 689,155 and 1,728,823 fitting parameters that show only a slightly lower error of 105 meV and 98 meV, respectively, which is still almost twice the error of the SchNet+H with 56 meV. Panels b to d show a scan along the bending mode of the molecule with zoom-ins to highlight the accuracy of the SchNet+H model compared to the MS model.

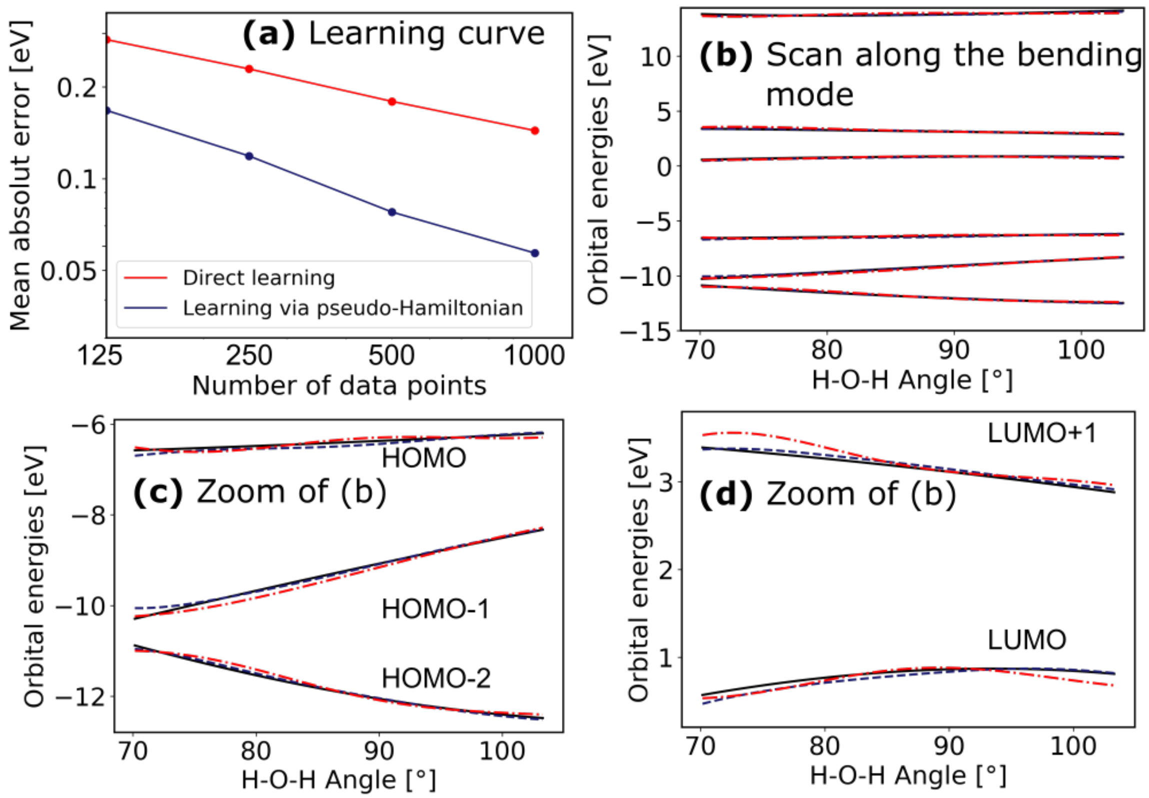

FIG. S1. (a) Learning curves of the multi-state and pseudo-H models, i.e., the error averaged over two ML models as a function of training set size for 15 eigenvalues of water. (b) A scan along the bending mode of the molecule shows the closest molecular orbitals around the HOMO and the LUMO with zoom-ins in panels (c) and (d), comparing the different models to the reference method. Learning curves show the slightly better learning efficiency and lower offset of the SchNet+H model.

S3. PERFORMANCE OF ML MODELS ON EIGENVALUES OF ETHANOL, QM7-X, QM9, OE62 AND GW5000 MOLECULES

Table S2 lists the MAEs and RMSEs of the models trained in this work on orbital energies of H_2O, ethanol, and the molecules in the QM7-X, QM9, OE62, and GW5000 data sets. For better comparison, we report errors of models that were previously used to fit one or more orbital energies of these data sets.

Scatter plots showing the predicted orbital energies for ethanol and the molecules in the QM7-X and QM9 data sets (using the model that predicts 30 eigenvalues) are shown in Figs. S2a-c, respectively, for the first 10,000 randomly mixed molecules within the test set. In addition, the worst predicted eigenvalues are shown in dark blue with the corresponding molecule within the plot. As can be seen, the worst predicted molecule in the QM9 data set (panel b) is a complex system with a four-membered ring attached to a five-membered ring. The two rings have an angle of almost 90 deg. The worst predicted molecule in the QM7-X data set has a CH_3 fragment and a highly distorted structure, which is energetically highly unfavourable.

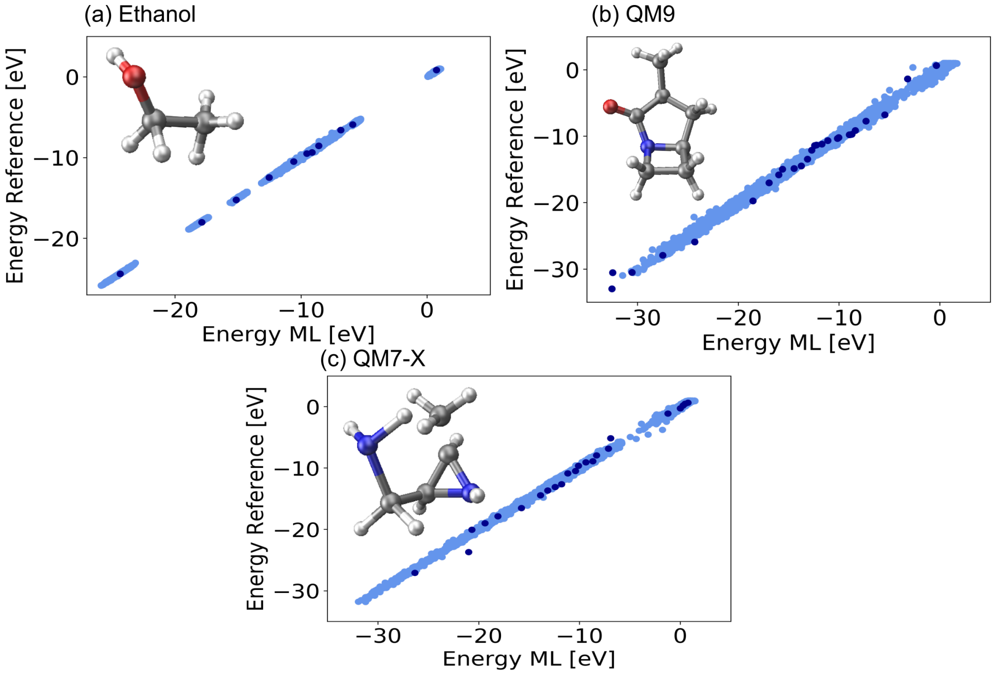

FIG. S2. Scatter plots showing the correlation between the predicted eigenvalues and the reference eigenvalues for 10,000 randomly selected molecules inside of the following training sets: (a) ethanol, (b) QM9, and (c) QM7-X. In addition, the worst prediction of the whole test set is shown with the molecular structure related to the worst predicted orbital energies shown in dark-blue.

To further support the findings of Fig. 2 in the main text, we plot the orbital energies and the diagonal matrix elements along an alchemical reaction coordinate (panel a) as predicted by an ML model trained on the OE62 data set in Fig. S3. As can be seen, the orbital energies are non-smooth functions which show avoided crossings across chemical compound space, whereas the diagonal matrix elements are allowed to cross and are smoother functions, even though spikes are visible in contrast to the configurational coordinate shown in Fig. 2 in the main text. In addition, the pseudo-Hamiltonian (pseudo-H) matrix elements are plotted

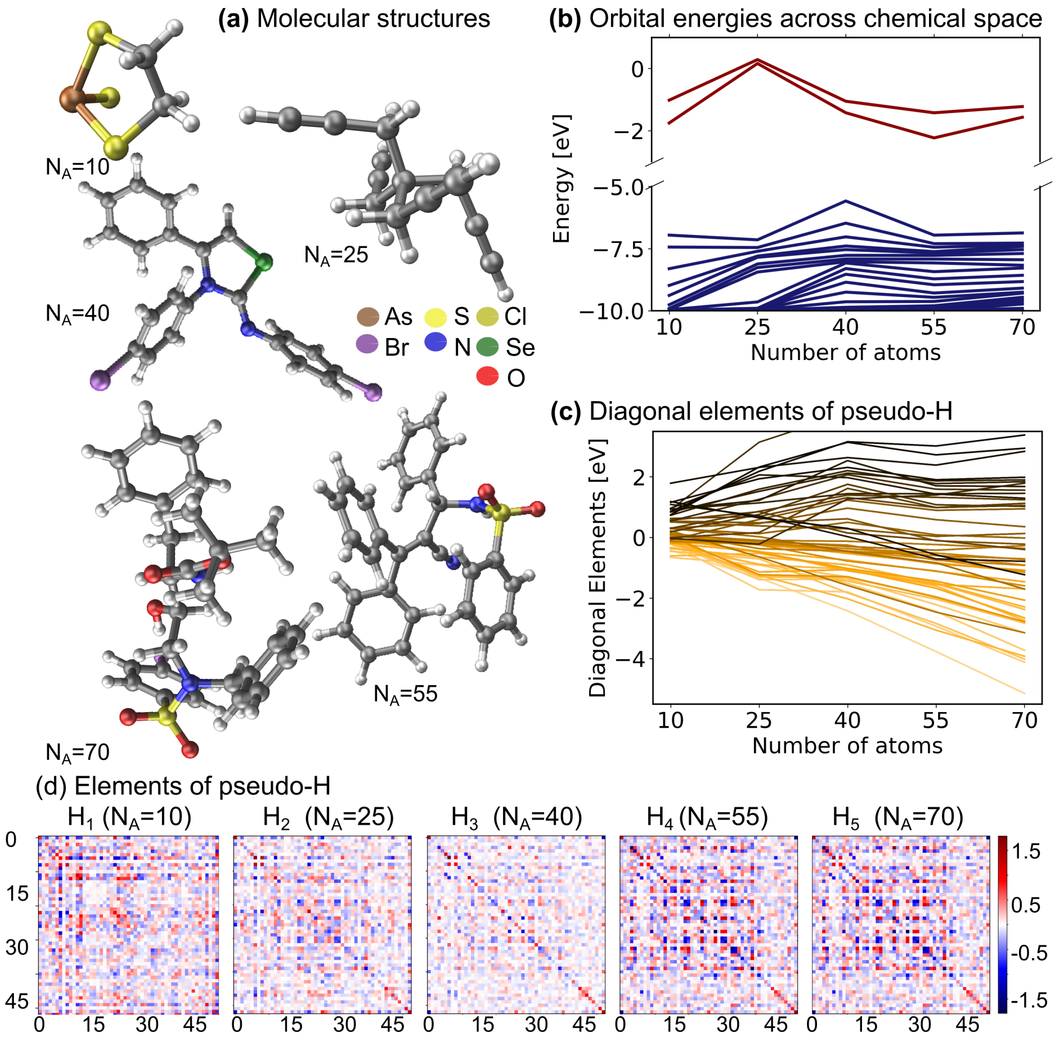

FIG. S3. Molecular orbital energies and pseudo-Hamiltonian (pseudo-H) diagonal elements are plotted (b and c, respectively) along (a) a reaction coordinate of different molecules with different number of atoms and elements using a ML model trained on the OE62 data set. (d) The pseudo-H matrices along the same reaction coordinate are shown. N_A is the number of atoms in a system.

in panel d for each molecule. It can be seen that the matrices are densely populated and become diagonally dominant for larger molecules (from left to right).

The OE62 data base contains molecules of high chemical complexity. Analysis of our model on the whole training set shows that some molecules cannot be predicted reliably. The average mean squared error (MSE) of all fitted orbital energies and the maximum MSE of the model on each data point of the whole training set is shown in Fig. S4a. As can be seen, some molecules are predicted with an extremely enlarged error and can be considered as outliers. These outliers, i.e., 18 data points, are shown with an in increasing mean root MSE (RMSE) on all orbital energies in panel (b) to assess the overall performance of the model on all orbital energies of these data points. As can be seen, the molecules with the largest model errors (17 and 18) contain bicyclic groups and contain many atoms. Another exemplary system is number 5, which contains an 8-membered cage that consists only of nitrogen atoms in the center. Molecule 13 is an example of a smaller system with

heteroatoms and of unusual composition. To investigate the influence of these systems on the training, the models are retrained without these data points and the accuracy of the models is assessed. As the model performance is not influenced, i.e., the MAE is the same and the RMSE differs slightly (0.21 eV instead of 0.19 eV), the models trained on all data points are used for further analysis. Even when the outliers for one model are removed, there are outliers that cannot be predicted accurately.

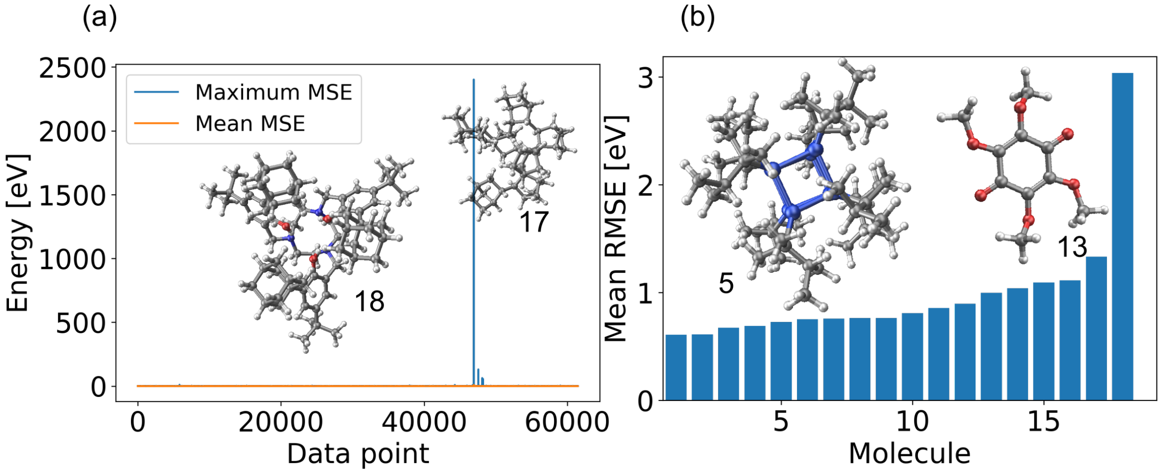

FIG. S4. (a) Maximum and mean orbital energy model MSE on the whole OE62 training set. Some outliers with an error larger than one third of the mean maximum RMSE and its standard deviation are sorted out and (b) the respective mean RMSE in increasing size to assess the overall performance on all orbital energies of the system.

The spectral shifts of the orbital energies of the molecules in the OE62 data set due to correction by perturbation theory are shown in Fig. S5b and are in agreement with the spectral shifts obtained in the GW5000 data set for which reference values exist.[8] To allow an assessment of the accuracy of the predicted values for which no reference values are available, a second Δ-ML model is trained for the differences of Kohn-Sham DFT values and quasiparticle energies and the differences due to implicit solvation. Only molecules whose values are predicted with a difference smaller than the MAE of the two trained models are considered trustworthy and are used for the analysis. In this way, 5661 (4592) quasiparticle predictions (orbital energies with implicit solvation) are sorted out. On average, the molecules sorted out contain about 75 atoms and the largest molecule is 174 atoms in size, while the remaining molecules contain on average 40 atoms and the largest molecule classified as trustworthy has 78 atoms. The GW5000 data set contains molecules that average 40 atoms in size and only 107 molecules in the training set contain more than 78 atoms, which we consider to be insufficient data to train a reliable model for systems of this size.

Furthermore, panel a shows the correlation of the HOMO orbital energies and the LUMO orbital energies of PBE0, G0W0@PBE0, and PBE0(H_2O). As can be seen from the light and dark red data points, the HOMO and LUMO energies of PBE0 calculated in the gas phase and with an implicit solvation model for water are not strongly different from each other and show a linear relation. A linear relation is also found when comparing the HOMO (dark blue) and LUMO energies (light blue) of PBE0 and G0W0@PBE0. However, as expected, the values do not lie on the diagonal with the HOMO values shifted towards lower energies

and the LUMO values shifted towards higher energies.

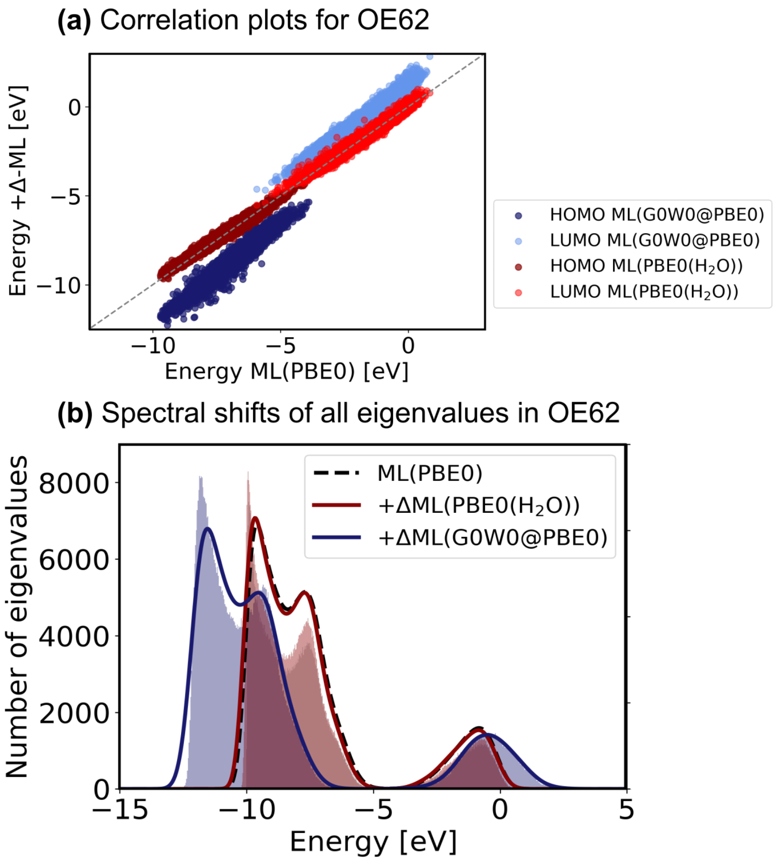

FIG. S5. The SchNet+H model for PBE0 eigenvalues and the Δ-ML models used to predict the differences of PBE0 to G0W0@PBE0 and PBE0(H_2O) eigenvalues for the whole 62k data set. (a) A linear correlation between the HOMO and LUMO eigenvalues of PBE0 and G0W0@PBE0 and PBE0(H_2O) can be found. (b) Gaussian functions with a width of 0.5 eV are placed on the eigenvalues and are summed up to show the trend of the spectral shifts of the molecules in the 62k data set. The shaded areas are obtained from a histogram analysis where the whole energy range of the spectrum is divided into 500 parts and orbital energies within a given energy range are grouped. The y-axis on the left refers to the number of eigenvalues within a given energy range for the training set.

Training set	ML model	Training points	Validation points	Test points	# ε [eV] (energy range)	MAE (RMSE) [eV]
Ethanol	SchNet+H	25k	1k	4k	$\leq$12 (-54 - 3)	0.05 (0.07)
Ethanol	SchNOrb*[11]	25k	500	4.5k	all	0.48
Ethanol	SchNOrb*[11]	25k	500	4.5k	12 ($\approx$ -54 - $\approx$ 3)	$\approx$ 0.017
QM9	SchNet+H	10k	1k	97.7k	$\leq$34 (-54 - 1)	0.23 (0.32)
QM9	SchNet+H	90k	9k	9.7k	16 (-54 - HOMO)	0.12 (0.16)
QM9	KRR[15]	32k			1 (HOMO)	0.086 (0.12)
QM9	1S-SchNet[16]	110k			1 (HOMO)	0.041
QM9	CNN[12]	$\approx$ 120k			16	- (0.23)
QM9	DTNN[12]	$\approx$ 120k			16	- (0.19)
QM7-X	SchNet+H	100k	10k	230.2k	$\leq$ 30 (-54 - 1)	0.15 (0.20)
OE62	SchNet+H	50k	5k	7k	$\leq$ 53 (-10 - LUMO+1)	0.13 (0.19)
OE62	KRR[15]	32k			1 (HOMO)	0.17 (0.24)
OE62	GNN[17]	32k			1 (HOMO)	0.15 (0.21)
OE62	GNN[17]	32k			1 (LUMO)	0.15 (0.21)
OE62	GNN*2[17]	32k			1 (HOMO)	0.13 (0.18)
OE62	GNN*2[17]	32k			1 (LUMO)	0.13 (0.18)
GW5000 (G0W0@PBE0)	MS-SchNet ΔML	4k	400	839	$\geq$52 (-10eV - LUMO)	0.16 (0.21)
GW5000 (G0W0@PBE0)	MS-SchNet ΔDFT	4k	400	839	$\geq$52 (-10 - LUMO)	0.028 (0.079)
GW5000 (PBE0(H_2O))	MS-SchNet ΔML	4k	400	839	$\geq$52 (-10 - LUMO)	0.11 (0.16)

TABLE S2. Test set errors on predicted eigenvalues of different training sets. Kernel Ridge Regression (KRR)[15], Convolutional Neural Networks (CNN)[12], Deep Tensor NNs (DTNNs)[12], Graph NNs (GNNs, *[2]with extended descriptors)[17], and SchNOrb models (*[1] model not trained on forces, only trained on energies)[1] are trained. G0W0@PBE0 and PBE0(H_2O) eigenvalues are predicted using a combination of a SchNet+H model trained on PBE0 eigenvalues of the OE62 data set and a Δ-ML model trained on G0W0@PBE0-PBE0 values from the GW5000 data set. ΔML indicates that the model is trained on the difference of PBE0 values obtained from the ML model, whereas ΔDFT indicates a model trained on the difference PBE0 reference values from DFT to G0W0@PBE0 values. The number of data points used for training, validation, and testing as well as the number of eigenvalues we trained are indicated along with the energy range that defines the number of eigenvalues for every molecule. The validation set The number of eigenvalues is related to the molecule that contains most eigenvalues within this energy range.

S4. SPECTRA PREDICTION OF UNSEEN MOLECULES IN ADDITION TO THE MAIN TEXT

In addition to the spectra predicted in the main text, additional excitation spectra of azulene-like molecules, polycyclic hydrocarbons, and azenes are shown in Figures S6 to S8, respectively. Noticeably, no molecule that is illustrated here, is contained in the GW5000 data set. All molecular structures were optimized at PBE level of theory using FHI-aims[18,19] in accordance with the reference data in the OE62 data set.[8]

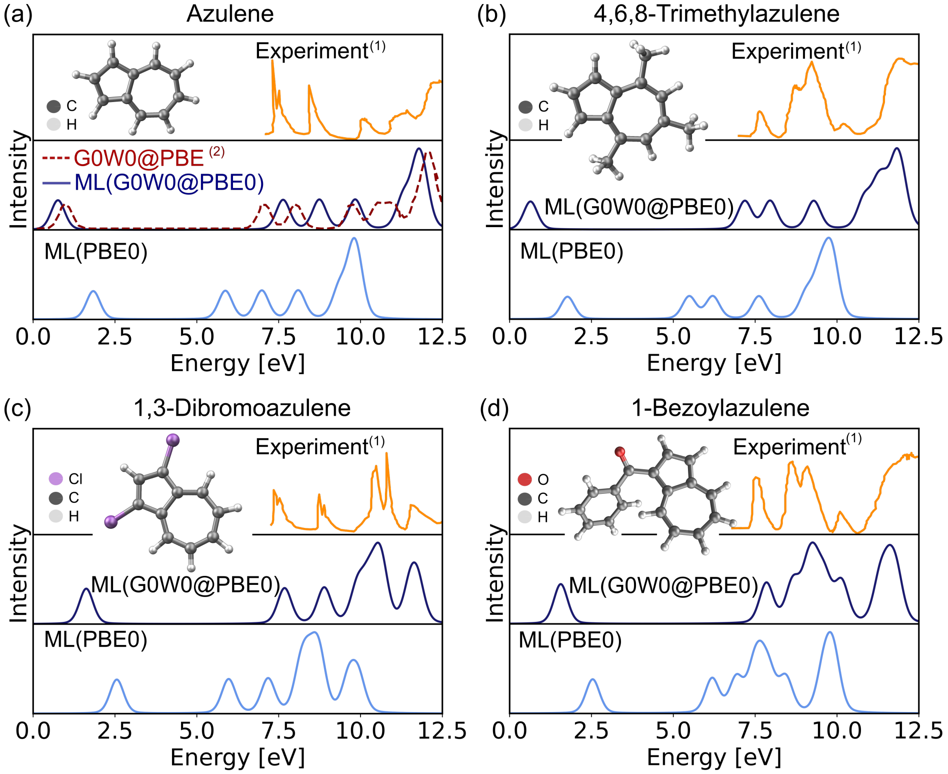

FIG. S6. Experimental and predicted photoemission spectra along with the LUMO (quasiparticle) orbital energies for PBE0 (G0W0@PBE0) for (a) azulene, (b) 4,6,8-Trimethylazulene, (c) 1,3-Dibromoazulene, (d) 1,3-Dichloroazulene, (e) 1,3-Dibenzoylazulene, and (f) 1-Benzoylazulene. (1)Experimental photoemission spectra are extracted from Ref. 20 (2)G0W0@PBE values for azulene are extracted from Ref. 21.

Fig. S6a shows the spectra of azulene at PBE0, G0W0@PBE, G0W0@PBE0 levels of theory with a comparison to experiment. The experimental data was extracted from published spectra.[20] The G0W0@PBE0 values predicted with ML match the experimental spectra better than the reference G0W0@PBE values. This effect can be attributed to the fact that the G0W0 method is non-self-consistent and heavily relies on the quality of the Kohn-Sham DFT orbital energies as starting point. All examples show that the energy gaps found with G0W0@PBE0 are considerably larger than those found with PBE0 and are in better agreement with experiment. Noticeably, the experimental spectra show a base line drift, which is an artifact due to the quality of the published spectra which date back to 1980.[20] Spectra created from the predicted resonances are obtained using a Pseudo-Voigt profile[22,23] to account for line broadening with a mix of 30% Lorentzian and 70% Gaussian with a width

of 0.5 eV.

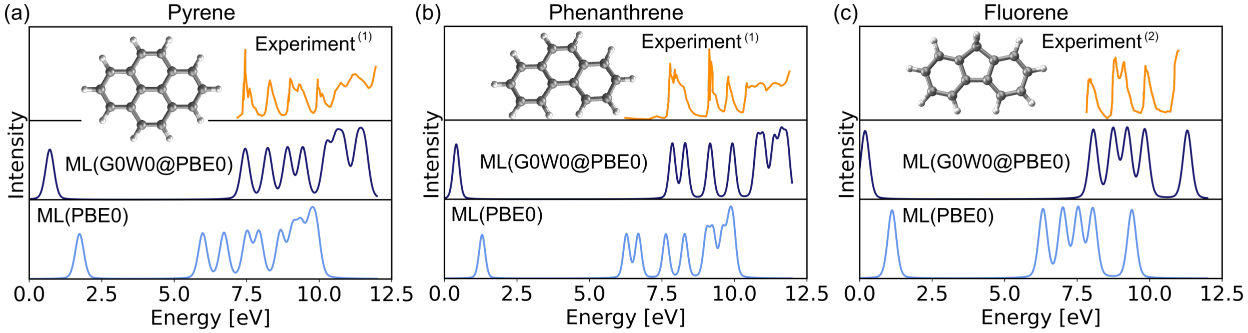

FIG. S7. Experimental and predicted photoemission spectra along with the LUMO (quasiparticle) orbital energies for PBE0 (G0W0@PBE0) for (a) azulene, (b) 4,6,8-Trimethylazulene, (c) 1,3-Dibromoazulene, (d) 1,3-Dichloroazulene, (e) 1,3-Dibenzoylazulene, and (f) 1-Benzoylazulene. A Pseudo-Voigt profile[22,23] with 30% Lorentzian and 70% Gaussian and a width of 0.3 eV is used. $^{(1)}$Experimental photoemission spectra are extracted from Ref. 24 and $^{(2)}$ Ref. 25.

Besides chrysene and perylene, which are already reported in the main text, pyrene, phenanthrene, and fluorene photoemission spectra are predicted and compared to experiment.[24,25] Those molecules are of special interest for novel functional organic materials. As can be seen, all spectra are in qualitatively good agreement to experiment. Further, the ionization potentials are almost perfectly reproduced with the ML models.

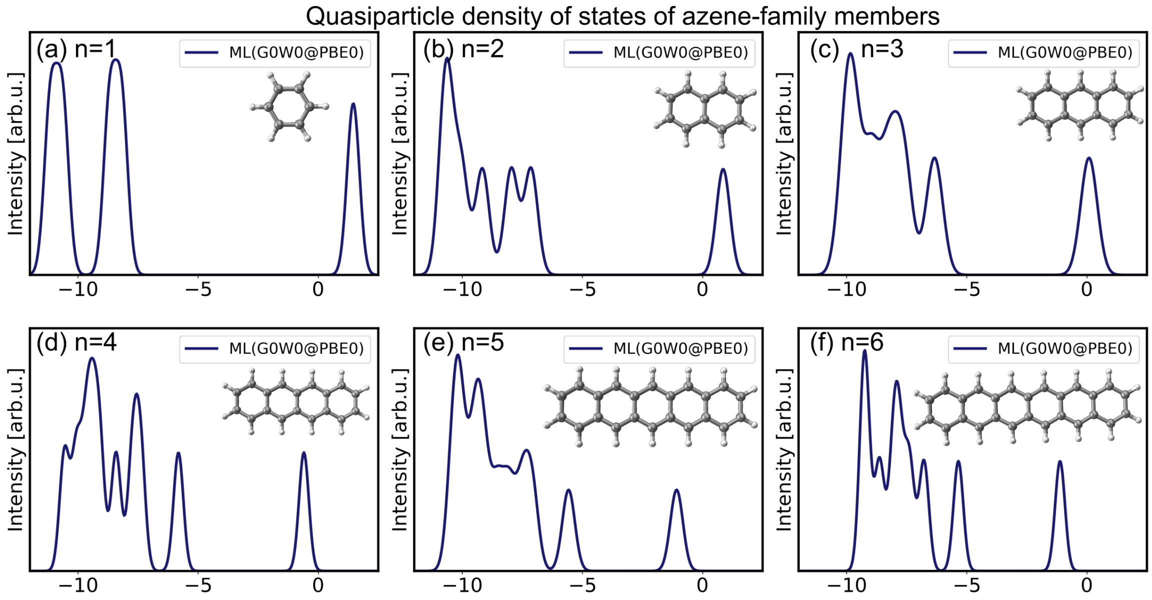

FIG. S8. Photoemission spectra predicted with ML models at G0W0@PBE0 quality.

Lastly, we plot the excitation spectra of azene at G0W0@PBE0 accuracy. It is known from literature[26] that the energy gaps are underestimated with G0W0 for these molecules, which can also be seen from the ML prediction in Fig. 4(e) in the main text. However, the trend exists that larger azenes lead to smaller HOMO-LUMO gaps. This trend can be reproduced with the ML models. Due to the shift in the spectral peaks of G0W0 with respect

to experiment, we only plot the ML predictions here using a Gaussian convolution of width 0.1 eV. Besides the shift, the spectra are in qualitatively good agreement with experimental values, that are summarized from different studies in Ref. 26.

REFERENCES

[1]K. Schütt, M. Gastegger, A. Tkatchenko, K.-R. Müller, and R. J. Maurer, "Unifying machine learning and quantum chemistry with a deep neural network for molecular wavefunctions," Nat. Commun. **10**, 1–10 (2019).

[2]M. Ernzerhof and G. E. Scuseria, "Assessment of the perdew–burke–ernzerhof exchange-correlation functional," J. Chem. Phys. **110**, 5029–5036 (1999).

[3]M. R. Raghunathan Ramakrishnan, Pavlo O. Dral and O. A. von Lilienfeld, "Quantum chemistry structures and properties of 134 kilo molecules," Sci. Data **1**, 140022 (2014).

[4]L. Ruddigkeit, R. van Deursen, L. C. Blum, and J.-L. Reymond, "Enumeration of 166 billion organic small molecules in the chemical universe database GDB-17," J. Chem. Inf. Model. **52**, 2864–2875 (2012).

[5]M. Gastegger, A. McSloy, M. Luya, K. T. Schütt, and R. J. Maurer, "A deep neural network for molecular wave functions in quasi-atomic minimal basis representation," J. Chem. Phys. **153**, 044123 (2020).

[6]F. Neese, "The ORCA program system," WIREs Comput. Mol. Sci. **2**, 73–78 (2012).

[7]J. Hoja, L. M. Sandonas, B. G. Ernst, A. Vazquez-Mayagoitia, R. A. DiStasio Jr, and A. Tkatchenko, "QM7-X, a comprehensive dataset of quantum-mechanical properties spanning the chemical space of small organic molecules," Sci. Data **8**, 43 (2021).

[8]A. Stuke, C. Kunkel, D. Golze, M. Todorović, J. T. Margraf, K. Reuter, P. Rinke, and H. Oberhofer, "Atomic structures and orbital energies of 61,489 crystal-forming organic molecules," Sci. Data **7**, 58 (2020).

[9]C. Adamo and V. Barone, "Toward reliable density functional methods without adjustable parameters: The PBE0 model," J. Chem. Phys. **110**, 6158–6170 (1999).

[10]M. J. van Setten, F. Caruso, S. Sharifzadeh, X. Ren, M. Scheffler, F. Liu, J. Lischner, L. Lin, J. R. Deslippe, S. G. Louie, C. Yang, F. Weigend, J. B. Neaton, F. Evers, and P. Rinke, "Gw100: Benchmarking g0w0 for molecular systems," J. Chem. Theory Comput. **11**, 5665–5687 (2015).

[11]M. Sinstein, C. Scheurer, S. Matera, V. Blum, K. Reuter, and H. Oberhofer, "Efficient implicit solvation method for full potential DFT," J. Chem. Theory Comput. **13**, 5582–5603 (2017).

[12]K. Ghosh, A. Stuke, M. Todorović, P. B. Jørgensen, M. N. Schmidt, A. Vehtari, and P. Rinke, "Deep learning spectroscopy: Neural networks for molecular excitation spectra," Adv. Sci. **6**, 1801367 (2019).

[13]J. Westermayr, M. Gastegger, and P. Marquetand, "Combining SchNet and SHARC: The SchNarc Machine Learning Approach for Excited-State Dynamics," J. Phys. Chem. Lett. **11**, 3828–3834 (2020).

[14]"Schnarc," Https://github.com/schnarc/SchNarc.

[15]A. Stuke, M. Todorović, M. Rupp, C. Kunkel, K. Ghosh, L. Himanen, and P. Rinke, "Chemical diversity in molecular orbital energy predictions with kernel ridge regression," J. Chem. Phys. **150**, 204121 (2019).

[16]K. T. Schütt, H. E. Sauceda, P.-J. Kindermans, A. Tkatchenko, and K.-R. Müller, "SchNet

– a deep learning architecture for molecules and materials," J. Chem. Phys. **148**, 241722 (2018).

[17]O. Rahaman and A. Gagliardi, "Deep learning total energies and orbital energies of large organic molecules using hybridization of molecular fingerprints," J. Chem. Inf. Model. **60**, 5971–5983 (2020).

[18]V. Blum, R. Gehrke, F. Hanke, P. Havu, V. Havu, X. Ren, K. Reuter, and M. Scheffler, "Ab initio molecular simulations with numeric atom-centered orbitals," Comput. Phys. Commun. **180**, 2175–2196 (2009).

[19]I. Y. Zhang, X. Ren, P. Rinke, V. Blum, and M. Scheffler, "Numeric atom-centered-orbital basis sets with valence-correlation consistency from H to Ar," New J. Phys. **15**, 123033 (2013).

[20]D. Dougherty, J. Lewis, R. Nauman, and S. McGlynn, "Photoelectron spectroscopy of azulenes," J. Electron Spectrosc. Relat. Phenom. **19**, 21 – 33 (1980).

[21]Y. Mei, C. Li, N. Q. Su, and W. Yang, "Approximating quasiparticle and excitation energies from ground state generalized kohn–sham calculations," J. Phys. Chem. A **123**, 666–673 (2019).

[22]M. Schmid, H.-P. Steinrück, and J. M. Gottfried, "A new asymmetric pseudo-voigt function for more efficient fitting of xps lines," Surf. Interf. Anal. **46**, 505–511 (2014).

[23]M. Schmid, H.-P. Steinrück, and J. M. Gottfried, "Erratum: A new asymmetric pseudo-voigt function for more efficient fitting of xps lines," Surf. Interface Anal. **47**, 1080–1080 (2015).

[24]M. S. Deleuze, "Valence one-electron and shake-up ionization bands of polycyclic aromatic hydrocarbons. ii. azulene, phenanthrene, pyrene, chrysene, triphenylene, and perylene," J. Chem. Phys. **116**, 7012–7026 (2002).

[25]P. M. Mishra, L. Avaldi, P. Bolognesi, K. C. Prince, R. Richter, and U. R. Kadhane, "Valence shell photoelectron spectroscopy of pyrene and fluorene: Photon energy dependence in the far-ultraviolet region," J. Phys. Chem. A **118**, 3128–3135 (2014).

[26]T. Rangel, K. Berland, S. Sharifzadeh, F. Brown-Altvater, K. Lee, P. Hyldgaard, L. Kronik, and J. B. Neaton, "Structural and excited-state properties of oligoacene crystals from first principles," Phys. Rev. B **93**, 115206 (2016).
